# Experience of illness with chronic singultus: a qualitative interview study

**DOI:** 10.1186/s13023-025-03619-1

**Published:** 2025-03-22

**Authors:** Marco Richard Zugaj, Claudia Busch, Andrea Züger, Jens Keßler

**Affiliations:** 1 Department of Anesthesiology, Pain Medicine Section, Heidelberg University, Im Neuenheimer Feld 131, 69120 Heidelberg, Germany; 2 Institute for History, Theory, and Ethics of Medicine, Justus Liebig University Giessen, Giessen, Germany

**Keywords:** Chronic singultus, Quality of life, Qualitative research

## Abstract

**Background:**

Chronic singultus lasting longer than one month is a rare disease. Due to its low prevalence, generating evidence about it is difficult. Patients with chronic diseases struggle with considerable restrictions in their quality of life. Chronic hiccups can lead to problems such as insomnia, anorexia, fatigue, exhaustion, weight loss, and depression. The aim of this study was to gain a better understanding of the quality of life of patients with chronic singultus and their experiences in contact with the healthcare system and with the general population.

**Methods:**

The data were collected using semi-structured interviews. The data analysis was carried out using qualitative structuring content analysis according to Kuckartz and Rädiker. Reliability was ensured by joint interprofessional evaluation of the interviews by experts, considering different perspectives.

**Results:**

Interviews from 20 patients with chronic singultus were analyzed. Analysis yielded 43 categories that could be assigned to five main topics. The disease burden of the patients was high. In addition to physical symptoms such as concomitant gastroenterological symptoms, shortness of breath, and fatigue, psychosocial consequences such as shame, social withdrawal, anxiety, depression, and even suicidality led to reduced quality of life.

**Conclusions:**

Ignorance and helplessness among healthcare stakeholders in the case of chronic singultus could lead to a marginalization of the disease and patients. Referring patients to a center with the appropriate expertise can help to avoid underuse, overuse, or misuse of healthcare. Therefore, the awareness of the disease among stakeholders must raise.

**Supplementary Information:**

The online version contains supplementary material available at 10.1186/s13023-025-03619-1.

## Background

Hiccups (singultus) is a widespread and little understood phenomenon [1, 2]. Hiccups are classified as transient (< 48 h), persistent (48 h − 1 month) or chronic (> 1 month) depending on their duration [3]. The afferent path of the reflex arc consists of the vagus and phrenic nerves. The efferent path consists of the phrenic nerve (C3-5), which innervates the diaphragm, the plexus branches to the scalene muscles (C5-7), the recurrent laryngeal nerve to the glottis and the intercostal nerves (T1-11), which innervate the intercostal muscles [2–4]. Gama-aminobutyric acid (GABA) and dopamine act as central neurotransmitters for this reflex. When the hiccup reflex is triggered, there is a synchronized contraction of the inspiratory thoracic muscles and the diaphragm. This is followed within 35 milliseconds by an abrupt closure of the glottis, which produces the typical “hiccup“ [2, 5]. There are no conclusive findings on how this reflex is triggered, but it can be assumed that any mechanism that irritates or damages the components of the reflex arc can trigger a hiccup [2].

Transient hiccups are well known to everyone, but chronic hiccups only affect a very small proportion of the population. Yet, the prevalence of chronic hiccups in the general population has not been measured. However, the incidence of hiccups in hospitalized patients was 55 in 100,000 in a retrospective analysis [6]. Around 50% of these were thought to have had a persistent singultus; none had chronic singultus. Especially chronic hiccups can lead to problems such as insomnia, anorexia, fatigue, exhaustion, weight loss, depression, unnecessary surgery, and even death [2, 7, 8]. Chronic hiccups occur more frequently in patients over the age of 50 and with underlying malignant diseases [6]. Often, no treatable etiology can be determined [9, 10]. To rule out known causes of hiccups, further diagnostics may be needed after taking a medical history: laboratory chemical tests, thoracic and abdominal CT, gastroscopy with pH examination, neurocranium MRI, bronchoscopy or toxicological screening [2]. If no specific causes can be found and eliminated, drug therapy with proton pump inhibitors is usually the next step in the treatment algorithm [11]. First-line drugs for further empirical therapy are baclofen [12, 13] and gabapentin or pregabalin [14]. Non-pharmacological therapies include hypnosis, acupuncture, nerve blocks or nerve stimulation [2]. The treatment options are based only on case reports [8, 15–17].

Access to experts and specialized treatment centers is a major problem for all rare diseases, as many doctors and healthcare facilities do not have sufficient experience or expertise [18]. This leads to delays in diagnosis and inadequate care. Lack of awareness and knowledge about rare diseases in the population leads to stigmatization, social isolation, and psychological suffering for patients [18]. Studies on rare diseases are usually too costly for individual research groups and impractical for industry due to the small number of cases and the geographical distribution of patients [18, 19]. One solution could be the collaboration of several research institutions from different regions [19].

The challenges of daily life with chronic diseases are multidimensional. First, there are physical impairments such as pain, fatigue, and difficulty with daily activities [20]. Psychological burdens such as anxiety, depression, and stress can severely impair quality of life, make it impossible to cope with the illness, and lead to social isolation [21, 22]. Financial burdens arise from the costs of medical assessments, medication, and other health-related expenses. At the same time, restrictions occur in the patient’s work life, which can lead to financial losses and job insecurity [20].

The experience of illness of patients has been well studied qualitatively and quantitatively for many chronic diseases [23]. However, generating evidence about chronic singultus is difficult due to its low prevalence [1, 12, 24, 25]. So, this study aims to better understand the experience of patients with chronic singultus, their quality of life, and their experiences in contact with the healthcare system and the general population.

## Methods

In addition to quantitative methods such as questionnaire surveys, qualitative methods play an increasingly important role in researching complex, multidimensional phenomena such as chronic diseases [26]. Also, qualitative methods appear to be particularly suitable for researching the health-related quality of life of people affected by rare diseases [19]. The aim of these methods is to capture the patients’ internal perspective and to explore the subjective attribution of meaning with regard to their experience of illness. Qualitative methods enable a change of perspective, a triangulation and contrasting of the results of quantitative research, and help to discover new research approaches.

### Ethics

This study was reviewed by the Ethics Committee of the Medical Faculty of Heidelberg (S-383/2023) and registered with the German Clinical Trials Registry (DRKS00032444).

### Recruitment

Patient recruitment took place between August 2023 and January 2024 at the Center for Chronic Singultus at Heidelberg University Hospital. This is a special outpatient clinic under the umbrella of the Center for Rare Diseases. Patients were made aware of the study via a poster in the center’s waiting area. In addition, patients throughout Germany receive telephone consultations from doctors at the Center for Chronic Singultus. After their scheduled consultation these patients were asked about their interest in participating in the study by the attending physician. Patients interested in participating in the study were informed in detail about the study by telephone by the study personnel and received written informed consent by post.

### Patient eligibility

The inclusion criteria were: patients with chronic singultus, age 18 or older, treatment at the Center for Chronic Singultus at Heidelberg University Hospital. The exclusion criteria were: lack of legal competence to consent or insufficient knowledge of German language.

### Sampling strategy

The aim of the sampling strategy was to achieve the greatest possible variation and heterogeneity and to relate the findings to the existing research literature. Therefore, the following criteria for the sample selection were defined in advance on the basis of the theoretical background: diverse ages and social milieus, different genders, diverse severity of illness, and disturbed or undisturbed relationship with the practitioners. Sampling was carried out by the treating physician (CB) and the study director (MRZ) using deductive sampling (dependent on prior theoretical knowledge). The selection of the sample was based on entries in the patient file. The sample should be large enough to achieve theoretical saturation and to find a sufficient number of contrasting cases.

### Data collection

Qualitative semi-structured guided interviews enable openness, structuring, and specification at the same time [27]. The interview guide was developed based on recommendations from specialist literature [28], the research question, and discussions within the research team. Piloting was conducted only within the research team. The guide contained narrative-generating questions intended to encourage patients to think about their own experiences of illness. Specifically formulated guiding questions, targeted follow-up questions, and the use of the problem-oriented prior knowledge described above supplemented the open narrative-generating questions. As an example: The question “What do they think? How can we help patients better in the future?” addressed the patient’s subjective beliefs and enabled an open exchange about barriers and facilitators (see online supplementary material 1 for an English translation of the interview guide). All interviews were conducted by the first author (MRZ), a male, specialist in anesthesiology and pain therapist, who is experienced in qualitative pain research and regularly attends supra-regional methods workshops. There was no treatment relationship between the interviewer and the study participants. Prior knowledge about the individual patients was limited to the categories of the sampling strategy. All interviews were conducted in German and by phone.

### Data preparation

The interview was recorded with a digital recorder (Philips DPM6700; Philips; Hamburg, Germany) and then transcribed verbatim by a member of the research group using software and hardware (Philips dictation and playback software SpeechExec 10; Philips; Hamburg, Germany). The transcription was carried out consistently according to established transcription rules [29]. Data protection, pseudonymization, and anonymization of the raw data are carried out in accordance with an audited data protection plan [30].

### Data analysis

The individual interviews recorded in text form were analyzed using the content-structuring qualitative analysis method according to Kuckartz and Rädiker [29, 31] as a focused interview analysis [29]. The analysis was carried out using data analysis software (MAXQDA Analytics Pro Training; VERBI Software; Berlin, Germany).

The content analysis was carried out between February and March 2024, considering both inductive (newly generated from the material) and deductive (based on previous theoretical knowledge) approaches simultaneously. By analyzing individual cases, the aim was to achieve a holistic investigation of lifeworld experiences by analyzing individual cases [31]. The evaluation was carried out in a predefined six-stage process (online supplementary material 2).

### Reliability of the data analysis

Various techniques were used to increase the credibility of the data analysis. The interviews were jointly evaluated by three co-authors: MRZ, CB (female, specialist in anesthesiology, supervising physician at the Center for Chronic Singultus, inexperienced in qualitative research), and AZ (female, Doctor in cultural studies, expert in qualitative social research). A system of categories was established by consensus and differentiated in a second step. The empirical data was coded according to written coding rules [29]. Quality criteria for categories were defined at the beginning and consistently applie [29]. A small-step audit trail was created so that reviewers could follow individual phases of study planning, data acquisition and data analysis. The research report was prepared in accordance with the “Standards for Reporting Qualitative Research” guidelines [32].

### Translation of empirical data with AI

Even when transcribing raw qualitative data, information that is conveyed in the intonation or facial expressions and gestures of the speaker can be lost. In addition, phrases and implications of what is said that are recognizable to native speakers can be lost in translation. The authors have therefore made a special effort to translate the empirical data into English with the subtext largely preserved. Artificial intelligence (DeepL; DeepL SE; Cologne, Germany) was also used for this purpose. After using this service, the authors reviewed and edited the content with the help of native speakers. The authors therefore take responsibility for the content of the publication.

## Results

### Patient characteristics

Interviews with 20 patients with a diagnosis of chronic singultus (R06.6) were included in the qualitative analysis. Interviews lasted between 20 and 40 min. A heterogeneous sample was selected (Table 1). The age of the study participants was between 20 and 90 years. The duration of the disease ranged from a few months to many years. The intensity of the disease ranged from “remission under therapy” to “most severe burden due to continuously occurring hiccups”.

**Table 1:** Characterization of the study sample

Interview	Age	Gender	Income	Place of residence	Onset of symptoms
1	46–50	M	< 50.000€	Small town	2018
2	60–65	F	50.000 to 100.000€	Big city	2022
3	55–60	F	< 50.000€	Small town	2023
4	30–35	M	> 100.000€	Village	2019
5	35–40	F	< 50.000€	City	2021
6	60–65	F	< 50.000€	City	2022
7	55–60	F	50.000 to 100.000€	Big city	2018
8	20–25	F	< 50.000€	City	2020
9	81–85	M	< 50.000€	Big city	2021
10	65–70	M	< 50.000€	Village	2006
11	61–65	M	< 50.00€	Small town	2018
12	26–30	M	> 100.000€	Big city	2023
13	36–40	F	< 50.000€	Village	2022
14	36–40	M	50.000 to 100.000€	City	2020
15	76–80	M	< 50.000€	Big city	1992
16	71–75	M	50.000 to 100.000€	City	2019
17	86–90	M	< 50.000€	Small town	2022
18	76–80	M	< 50.000€	Village	2022
19	76–80	M	< 50.000€	City	2022
20	76–80	M	< 50.000€	Big city	2021

### Main categories

Over 10 h of interview material were analyzed. Seven hundred ninety-five segments were assigned to 43 categories. These were grouped into 5 main categories: change in quality of life, disease framing, barriers and support outside the healthcare system, stakeholders in the healthcare system, and medical history.

### Theme 1: change in quality of life

Patients reported that the disease severely impaired their quality of life. Accompanying symptoms (Table 2) that occurred in addition to chronic hiccups were particularly burdensome. Gastroenterological concomitant symptoms such as nausea and vomiting were reported by 12 patients. Some patients used forced vomiting to interrupt the hiccups. Other life-limiting concomitant symptoms were shortness of breath, fatigue, and sleep disturbances.

The emotional burden of the disease was described as severe. Frequently mentioned emotions were anxiety, aggression, dejection, and shame (Table 2). Fifteen patients reported social withdrawal after the onset of the disease. Communication with other people was impaired. In a third of patients, shame for the hiccups was a reason for social withdrawal.

Patients reported a change in their self-image and their public image as a result of the chronic illness (Table 2). The loss of body control was described as drastic. Patients with high performance demands (competitive athletes, bodybuilders) described a severe drop in performance. Patients who had not previously had any restrictive experiences of illness had to come to terms with the change in perspective of a chronically ill person.

**Table 2:** Main category “change of quality of life” – Subcategories: (A) accompanying symptoms, (B) emotional burden, and (C) change in self-image with sub-subcategories and examples of quotation

**A) Accompanying symptoms - Sub-subcategory:**	ID: Examples of quotations
Gastrointestinal symptoms	B6: And yes (.) then (.) I realized […] that if I stick a finger down my throat and try to gag […] then I always had peace with the hiccups for an hour or two. And now I do this regularly.
Sleep disorder	B7: The problem is always that you can’t fall asleep. Because of these stupid hiccups, I’ve more or less migrated to the couch and I lie down there because my wife is also working.
Fatigue	B1: What bothers me most is this exhaustion.
Shortness of breath	B17: Anyway, I couldn’t breathe, I really thought I was going to suffocate.
**B) Emotional burden - Sub-subcategory:**	
Shame	B18: […] on the bus and it was always full of people. I always thought oh God or even on the train. I always thought they would all notice that I had the hiccups. That was a bit embarrassing for me […].
Anxiety	B2: That’s the most unpleasant thing about this. And it’s quite a burden for me. I’m directly afraid of falling over here in the kitchen or something, yes. It’s not like I’m scared to death, but it’s true that I’m terrified.
Aggression	B7: And then of course I get angry and aggressive inside because I’m annoyed with myself, because I always think, why do you have to have the hiccups so badly right now that you can’t communicate properly?
Dejection	B10: Because I had the hiccups, I couldn’t talk to people. I withdrew a bit. And then sadness set in. I sat down in a corner and just listened.
**C) Change in self-image - Sub-subcategory**	
Change in self-image	B12: And so my body has always functioned optimally, to put it bluntly. And this break with the fact that the body becomes kind of weak. (.) How should I put it? (.) It was something very new and I had to learn to deal with it psychologically. And I don’t really want that and I’m currently looking for a solution with the [specialist] and then to bring that back into everyday life.
Changed public image	B1: You’ve also lost a bit of self-esteem because you can no longer take part in many things, because you’re no longer part of working life. Work meant a lot to me.

### Theme 2: disease framing

This main category subsumed beliefs about causes, subjective attribution of treatment successes, affective evaluations of the illness, and wishes for the future (Table 3). Some patients reported a subjective connection with a SARS-CoV-2 infection, vaccination, or the frightening pandemic situation. In addition to recovery, patients also expressed the wish for visibility of their suffering as personal wishes for the future. Patients wished more social and health policy acceptance and support in coping with the disease. In addition, patients wished for more networking among doctors and rapid referral to centers with experience in treating their rare disease.

**Table 3:** Main category “disease framing” - Subcategories and examples of quotation

Subcategory	ID: Examples of quotations
SARS-CoV-2	B19: I had a corona infection and since then I have suffered from recurring constant hiccups.
Cause conviction	B18: At some point I realized that when I eat something sweet, it always triggers something.
Affective evaluation	B7: “Oh come on, it’s not that bad. There are worse diseases.” But then you think to yourself, what could be worse? Sure, if someone has cancer or something else, or is paralyzed from the neck down, that’s worse, of course. But at that moment, you only see yourself somewhere.
Subjective attribution of therapy successes	B13: We also try to do a lot with relaxation. We go to the sauna three times a week, which we have in the village, just to get some warmth into me. Because I notice that when I keep warm or am in the sauna, I feel really good.
Wishes for the future	B17: […] to make it clear to all doctors that hiccups and chronic hiccups are at least really a disease and can lead to severe suffering for the patient. B15: […] specialists (…) show the commitment to cross-connect. In other words, if I as a specialist find that I’m not getting anywhere, I take the initiative myself and (…) contact others […] to help my patients. B7: No, I would just think it would be good if there was a support group somehow. To be able to talk to people who have the same thing […].

### Theme 3: barriers and support outside the healthcare system

The patients independently searched the internet for information about their disease. Some were able to find our Center for Rare Diseases. Others had mainly found home remedies against transient hiccups that they tried without success. Information about chronic singultus is rare on the internet. Nocebo effects during the research were not reported. Mixed experiences were noted in relation to peers. A common theme was the mixture of sympathetic advice but also ignorance and lack of understanding of the condition. Many patients were of retirement age. Working patients had different experiences of integrating the condition into their everyday working lives. Some patients were no longer working due to the disease, while others continued to work despite the symptoms (Table 4).

**Table 4:** Main category “barriers and support outside the healthcare system” - Subcategories and examples of quotation

Subcategory	ID: Examples of quotations
Workplace	B19: At work, where I sit in meetings all day and am with people, it was very stressful because I always tried to suppress it.
Partnership	B13: That has also affected the relationship, although my husband always stands behind me, but sometimes it’s so stressful that he himself sometimes says he can’t take any more. And we have to find a way out of this now.
Family	B11: Oh, I was at the bottom, I tell you. Honestly. I was really, really down. And that’s why I spend a lot of time with my family, with my parents. That also put a strain on my family
Internet	B15: But of course - I can only speak for myself - the temptation is great to see what the media and [internet]portals or similar say about this hiccup phenomenon. But in the end, that didn’t give me anything that would have helped me on my path to healing.
Friends and acquaintances	B12: Yes, so there was a lot of surprise that it was so extremely intense. Hardly anyone knew it like that. I don’t know anyone who was like: “Ah yes. I know that.” Not that at all, but less incomprehension. More astonishment that something like that exists and helplessness. (Laughs) I mean, of course, friends, acquaintances and family want to help you, but they don’t really know the best way to do it because they simply don’t know any who can help.

### Theme 4: stakeholders in the healthcare system

Patients reported ignorance and helplessness of helpers on the part of practitioners. There were repeated reports of marginalization by practitioners and by health and pension funds. Patients reported difficulty getting appointments with specialists. They also reported personal barriers to engaging in non-drug therapies, such as breathing therapy or relaxation techniques (Table 5).

**Table 5:** Main category “stakeholders in the healthcare system” - Subcategories and examples of quotation

Subcategory	ID: Examples of quotations
Marginalization	B17: I also went to hospital a second time after I couldn’t breathe. And when I was admitted, they said: “Sit down in the waiting room. Fifteen minutes later, a chief nurse came in and introduced herself. She was the same one I’d seen before when I was in hospital for treatment. She said to me: “Yes, I’ve looked at your records. It says you’re healthy. We can’t help you here. You have the hiccups and we can’t help you. You have to live with it. That can’t be changed. And we can’t treat it. You have to go home.” B8: With the hiccups, however, I often had the feeling that it was a bit like a side note: “Ah yes, hm.” They [doctors] thought about it for a moment: “Maybe you’ll be lucky and it will go away.” And then the topic was over because the people [doctors] themselves didn’t really have a clue and I think some of them [doctors] felt uncomfortable because they couldn’t tell the patient anything for sure.
Barriers	B10: I went to breathing therapy. It didn’t really do anything for me. It made me feel a bit strange. Why? Yes, I don’t know. Singing and what else, that’s not my thing.
Enabler	B7: That’s why I’m glad that I have my [family doctor], who really supports me and takes it seriously.
Therapy successes	B4: I was then told to take gabapentin, which I tolerated much better, at least initially at a fairly low dosage. This was then slowly increased and actually brought about a considerable improvement.

### Theme 5: medical history

Patients reported overuse and misuse of treatment. One patient reported a gastrectomy. One patient reported tonsillar abscess splitting “without anesthesia” which he associated with chronic hiccups and which was associated with severe pain and anxiety. Patients reported contact with speech therapists, acupuncturists, hypnotists, gastroenterologists, neurologists, visceral surgeons, pulmonologists, ear, nose and throat specialists, general practitioners, osteopaths, psychologists, and psychosomatic specialists. All patients used self-therapies such as diets or distraction methods for self-treatment. Drinking water was a common home remedy (Table 6).

**Table 6:** Main category “medical history” - Subcategories and examples of quotation

Subcategory	ID: Examples of quotations
Duration of illness	B11: Yes, it started at the end of 2018 and yes, I experienced it very badly. It went on until 2022. I had about 13 to 15 attacks a day and then I started to count the hiccups. Especially at night, when I went to bed, I got up to 350 hiccups. When I went to the [Center for Chronic Singultus] for treatment in 2022, the whole thing decreased incredibly.
Therapy experience	B13: Exactly, I was actually checked from head to toe via urine, stool, blood, ECG, MRI, CT. They did a neurological MRI to see if everything was okay neurologically. Then EEG, various sonographies of the bowel. They checked everything. So really once from head to toe and then when I told the doctor that my chest often hurts and that I haven’t been able to wear a bra for a long time because it always feels so tight, she referred me from the clinic to an orthopedist.
Home remedy	B18: Then I looked on the internet and saw that I was holding my breath, drinking water and thinking about what I ate for dinner last Good Friday, all that kind of stuff. So that didn’t help me at all.

## Discussion

The aim of this study was to describe the subjective illness experience of patients with chronic singultus. A qualitative study approach in the sense of a content-structuring interview analysis according to Kuckartz and Rädiker was chosen. The deductively (dependent on prior theoretical knowledge) selected patient collective had a range of disease burden. Various social milieus and various representative life stages were selected.

Research into chronic hiccups has so far taken a practitioner-centred perspective. Etiology and therapy in particular have been described in case reports and reviews [1]. There was a gap in the literature on the subjective perception of the patient and the psychosocial effects of the chronic disease, which we were able to explore with the interview design of our study.

Our data show that the quality of life of patients with chronic singultus suffers. In addition to the accompanying symptoms such as gastroenterological complaints, fatigue, and insomnia, primarily emotional aspects worsen patients’ quality of life. While other studies have indicated that chronic hiccups can lead to depression, anxiety, and exhaustion [7], our data show and approve potential mechanisms of action. These included shame - one of the most commonly cited psychosocial effects - resulting in social withdrawal and marginalization. This places the patient in a vicious circle that can exacerbate the psychosocial effects. With regard to marginalization, it must be emphasized that this is a multiple marginalization that is experienced not only among friends and family, but also among healthcare providers. This marginalization is often preceded by ignorance. The participants in this study reported that their doctors sometimes had no knowledge of the chronic singultus. The coordination of efforts was left to a courageous GP or even the patient’s or relatives’ own initiative. We already know that marginalization can lead to negative health outcomes; can cause stress, anxiety, or depression; and can make access to healthcare more difficult [33].

In addition to the factors mentioned above, the change in self-image is also related to the quality of life experienced. Our patients reported a loss of self-efficacy, performance, and confidence in their own bodies. We already know from other chronic diseases that those factors can increase insecurity [34, 35].

With regard to the alleged cause of illness, two patients mentioned a SARS-CoV-2 infection or vaccination. In principle, any irritation of the reflex arc could trigger chronic singultus. A few case reports described the coincidence of singultus (persistent - median 4,8 days) with a SARS-CoV-2 infection [36]. Whether the SARS-Covid-19 virus is also associated with an increased rate of chronic singultus could be the focus of further research.

Based on our results, it is clear that active efforts are needed to improve caretaking. An early connection to a specialized center could help to avoid overuse, underuse, or misuse of care. To do this however, the awareness of the disease among the stakeholders must be raised. Positive effects for both patients and payers can be anticipated. Furthermore, more data is needed to ensure evidence-based care and support. While a national registry collects enough data for evidence-generating research in other rare diseases [37, 38] such a registry is lacking for chronic singultus.

This study has several limitations that must be kept in mind. First, the number of cases in qualitative research is small and the sampling does not meet the criteria of random selection (deductive sampling, voluntary participation, bias of selection). Therefore, unlike quantitative research, qualitative research cannot claim generalization in the form of statistical representativeness [29]. In these studies however, generalization takes the form of an empirically based theory or the recognition of patterns and not the determination of statistical significance [29]. Second, due to the setting of the study adaptation of the participants’ statements due to social desirability cannot be completely ruled out. Third, the interviewer’s prior knowledge about the patients could have influenced the interview. Although the interviews were characterized by fundamental openness and non-judgement and the narrative flow was not interrupted.

## Conclusion

In conclusion, chronic singultus is an existential burden for those affected and is associated with a considerable reduction in their quality of life. Ignorance and uncertainty among healthcare providers can lead to a regrettable marginalization of the patients. However, the establishment of a national registry for chronic singultus patients would help to gather data on this rare disease, enabling treatment to be based more on reliable evidence rather than anecdotes and potentially improving the lives of those affected.

## Electronic supplementary material

Below is the link to the electronic supplementary material.

Supplementary Material 1

Supplementary Material 2

## Data Availability

The datasets generated and/or analysed during the current study are not publicly available following the data protection plan approved by our ethics committee, but are available from the corresponding author on reasonable request.
