# Supplementary material for: Experience of illness with chronic singultus: a qualitative interview study
Source: Orphanet J Rare Dis. 2025 Mar 22;20:141. doi: 10.1186/s13023-025-03619-1 (PMC11930004; doi:10.1186/s13023-025-03619-1)
Supplement: Supplementary file 1 — Supplementary Material 1 [file 13023_2025_3619_MOESM1_ESM.docx]

**Interview guide:**

**Effects of chronic hiccups on the quality of life of affected patients - a qualitative analysis**

| Setting | Uniform declaration before the interview | Icebreaker |
| --- | --- | --- |
| Pain outpatient clinic, university clinic, an assumption of strangeness cannot be credibly conveyed.  Goal: Create openness  Focused or semi-structured guided interview  Open questions first, three topics. Follow-up questions are possible in order to get to relevant aspects.  The interview always opens with the same explanations. Then the icebreaker demographic data.  The aim is to produce text.  Narrative-generating questions, with few presuppositions. | This interview is about your feelings, attitude and view of things.  They should tell their stories as freely as possible and are only given a rough direction.  There is no right or wrong. What is important is what YOU think. You will not be judged. Your statements are anonymous. Your statements have no influence on the therapy or are otherwise used outside the research.  We just need to be able to count on you to speak fully, **openly and truthfully.** | **Demographic data:**  Age:  Relationship status?  Place of residence (size)?  Level of education?  Annual income (< 50k, up to 100k, more |

| Leading question (narrative prompt) | Check - was this mentioned? Memo for follow-up questions - ask if necessary if not addressed by yourself. Adapt wording | Specific questions - Ask in this formulation at the end | Maintenance and control issues |
| --- | --- | --- | --- |
| Part 1: Biological/psychological bundle  Tell us about your hiccups story, from the beginning until today. In addition to the facts and figures, we are particularly interested in how you personally experienced the disease. | Symptoms:   - Hiccups - Tiredness - Sleep - Nausea - Shortness of breath - Control - Coping strategies - Body image - satisfaction   Emotions:   - Happy/sad - Shame - Aggressiveness/rage/balanced/fulfilled   Therapy so far:   - Home remedy - Medication - Supportive therapy - Operations | - Which symptoms affect you the most? | Can you tell us more about this?  What happened next?  What was that about...? |
| Leading question (narrative prompt) | Check - was this mentioned? Memo for follow-up questions - ask if necessary if not addressed by yourself. Adapt wording | Specific questions - Ask in this formulation at the end | Maintenance and control issues |
| Part 2: Social bundle  Why don't you tell us how dealing with other people has changed for you in connection with the illness? | Self-image:   - Clothing, appearance, self-confidence   Social support   - "good advice" - Hiccups -> Alcohol   Leisure time   - Social withdrawal - hobbies   Work   - Presenteeism/absenteeism - Conscience - Money (expenses for the illness)   Relationship   - Aggressiveness - Sleep of the partner   Medical treatment   - Stakeholders - Overuse - underuse - misuse | - Is enough/too little/too much being done to combat the disease? | Can you tell us more about this?  What happened next?  What was that about...? |

| Leading question (narrative prompt) | Check - was this mentioned? Memo for follow-up questions - ask if necessary if not addressed by yourself. Adapt wording | Specific questions - Ask in this formulation at the end | Maintenance and control issues |
| --- | --- | --- | --- |
| Part 3: Future  What do they think? How can we help patients better in the future? | Framing of the illness and how it is handled by the treating person   - Helplessness of the helpers - Stakeholder health   - Doctors   - Health insurance companies   - Therapists   - Information on   - Self-help groups - (surgery cures, diet cures, yoga cures, nothing cures, what relieves? acceptance?) | Can you think of anything else that has not yet been mentioned? | Can you tell us more about this?  What happened next?  What was that about...? |
