# Supplementary material for: Experience of illness with chronic singultus: a qualitative interview study
Source: Orphanet J Rare Dis. 2025 Mar 22;20:141. doi: 10.1186/s13023-025-03619-1 (PMC11930004; doi:10.1186/s13023-025-03619-1)
Supplement: Supplementary file 2 — Supplementary Material 2 [file 13023_2025_3619_MOESM2_ESM.docx]

Detailed presentation of the six-stage evaluation process of empirical data according to Kuckartz and Rädiker

| Step 1: Data preparation and exploration | This step involves intensive reading of the interviews and writing initial summaries and text memos. |
| --- | --- |
| Step 2: Deductive preliminary categorization | The first categories are formed deductively from the guideline and the prior theoretical knowledge of the investigators. |
| Step 3: Basic coding | The preliminary categories are used to analyze the interviews and sections of text are assigned (coded) to the categories. Further categories are defined and finally anchored in a fixed category system. |
| Step 4: Fine coding | A tabular listing of all text passages in a category allow a more in-depth analysis and differentiation into subcategories. |
| Step 5: Data analysis | Data of interest to the research question are selected and contrasted. |
| Step 6: Documentation | Checking the documentation of all individual steps. Writing a research report. Archiving the data in accordance with the data protection concept and advice on labor law from the responsible ethics committee. |
